# In Silico Guided Design of Metal/Semiconductor Photocatalysts: A Case of Cu-Modified TiO_2_ for Ciprofloxacin Degradation

**DOI:** 10.3390/ma16165708

**Published:** 2023-08-21

**Authors:** Marija Kovačević, Sanja Živković, Miloš Ognjanović, Miloš Momčilović, Dubravka Relić, Dragana Vasić Anićijević

**Affiliations:** 1 Vinča Institute of Nuclear Sciences—National Institute of the Republic of Serbia, University of Belgrade, Mike Petrovića Alasa 12-14, 11351 Belgrade, Serbia; marija.kovacevic@vin.bg.ac.rs (M.K.);; 2 Faculty of Chemistry, University of Belgrade, Studentski Trg 12-14, 11158 Belgrade, Serbia; dradman@chem.bg.ac.rs

**Keywords:** DFT calculations, photocatalysis, ciprofloxacin, rutile TiO_2_, degradation kinetics

## Abstract

(1) Background: An increasing use of pharmaceutics imposes a need for the permanent development of efficient strategies, including the tailoring of highly specific new materials for their removal from the environment. Photocatalytic degradation has been the subject of increasing interest of the researchers in the field. (2) Methods: This paper is focused on the investigation of the possibility to deposit a thin metal layer on a TiO_2_ surface and study its photocatalytic performance for the degradation of ciprofloxacin using a combination of theoretical and experimental methods. (3) Results: Based on the extensive DFT screening of 24 d-metals’ adhesion on TiO_2_, Cu was selected for further work, due to the satisfactory expected stability and good availability. The (Cu)TiO_2_ was successfully synthesized and characterized with XRD, SEM+EDS and UV-Vis spectrophotometry. The uniformly distributed copper on the TiO_2_ surface corresponds to the binding on high-affinity oxygen-rich sites, as proposed with DFT calculations. The photocatalytic degradation rate of ciprofloxacin was improved by about a factor of 1.5 compared to the bare non-modified TiO_2_. (4) Conclusions: The observed result was ascribed to the ability of adsorbed Cu to impede the agglomeration of TiO_2_ and increase the active catalytic area, and bandgap narrowing predicted with DFT calculations.

## 1. Introduction

An increasing use of antibiotic drugs worldwide is inevitably linked with raising pollution concerns [1,2]. Estimated average environmental water drug concentrations are between nanograms and micrograms per liter [3]. Due to the limited biodegradability of antibiotics and thus the limited ability of sewage treatment plants to remove them, they are ubiquitous and persistent according to their physico-chemical properties, thus leading to the hidden consequences of the chronic exposure and development of resistant microorganisms [4,5]. Efforts in resolving these issues impose a permanent need for developing novel techniques, and improving existent techniques, for their efficient and sustainable removal from the environment.

Photocatalytic degradation belongs to the group of advanced oxidation process methods (AOPs), and is widely investigated as a universal, efficient and environmentally friendly method for the elimination of various organic pollutants, including organic compounds (drugs, dyes and pesticides) and microorganisms (bacteria), from aqueous media [6]. The efficient photo-induced generation of hydroxyl radicals (·OH), as the principal oxidizing species [7], is of crucial interest for the performance of photocatalytic systems, in order to maximize the mineralization extent of organic compounds, minimize the generation of secondary byproducts and assure zero secondary waste [8,9].

Most commonly investigated photocatalytic materials include metal oxide semiconductors such as TiO_2_, ZnO, SiO_2_, Fe_2_O_3_, CdS and ZnS, due to their satisfactory stability, corrosion resistivity, availability and non-toxicity [10].

On the other hand, their photocatalytic performance is limited due to their tendance to agglomerate according to the high surface energy, large band gap (requires UV range light) and short recombination time of photogenerated electron–hole pairs [8,11].

Several strategies based on semiconductor modification by metals and metal oxides (doping, metal loading, core/shell systems and semiconductor combinations—introduction of heterojunctions) have been developed in order to overcome these issues [9,12,13]. Most of the studies in the field agree that a sophisticated design and highly precise control of material modification are crucial to achieve improved photocatalytic performances, as otherwise the heterostructural features can easily transform to recombination centers and further reduce charge carriers’ lifetime, induce catalyst surface clogging and reduce overall efficiency [14,15,16,17].

Single metal–atom and thin metal layer surface catalysts have been the subject of permanent but emerging interest of researchers, due to the possibility to obtain significantly different properties compared to bulk materials, and are made with a minimal consumption of expensive resources [18]. In order to efficiently tune their photocatalytic performances, it is necessary to enable the insight into their fundamental properties on the atomic level [19].

DFT calculations represent an efficient tool not only for the prediction of behavior of metal/semiconductor interfaces but also for the explanation of their structural and electronic properties up to the level of a single atom [20,21,22,23,24]. The ongoing development of catalytic descriptors [25] and calculation procedures to overcome present methodological drawbacks [26] widens the possibility to employ in silico experiments in the design of catalyst materials. However, despite extensive research, to our knowledge, there is still a lack of systematic studies that investigate metal thin layer formation and behavior on semiconductor surfaces.

**Figure 1:**
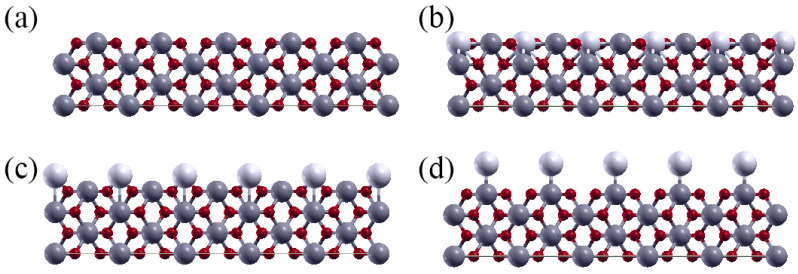
Structures of bare TiO_2_ (**a**) and (M)TiO_2_ with M on the *hollow* (**b**), *bridge* (**c**) and *top* (**d**) sites. Color code: Ti—gray, O—red, M—white.

In this study, a novel strategy for the preparation of thin layer metal/semiconductor photocatalysts based on DFT-guided design is presented and applied on TiO_2_ (rutile). An extensive DFT screening of d-metals’ binding on the TiO_2_ surface was performed in order to provide a systematic insight into the adhesion properties and predict the thin layer quality of the investigated metals. The aim was to identify a suitable metal to prepare a thin layer metal/TiO_2_ model system with an easily controllable structure, to be further studied experimentally and theoretically. Based on the screening results, the Cu coating was selected for TiO_2_ modification as effectively adsorbed, available and easy-to-prepare with the NaBH_4_ reduction method. The photocatalytic effectiveness of the prepared nanoparticles was evaluated towards the removal of the antibiotic drug ciprofloxacin (Figure 1 and Figure S1) in an aqueous suspension under UV irradiation. The improvement of the degradation rate with (Cu)TiO_2_ compared to bare TiO_2_ by a factor of 1.5 was noticed. The obtained improvement was discussed from the point of view of DFT insights and experimental findings, shedding new light on the photocatalytic properties of catalyst materials modified by thin layer metal deposition.

## 2. Materials and Methods

### 2.1. DFT Calculations

For DFT calculations, a pwscf code of the Quantum ESPRESSO package (version 6.6) was used [27]. Ultrasoft pseudopotentials based on GGA-PBE approximation [28] with a plane wave kinetic energy cutoff of 50 eV were implemented, while the charge density cutoff was 500 eV. Optimized rutile bulk parameters were *a* = 4.639 Å and *c* = 2.968 Å. In the DFT screening of the adhesion of different d-metals, the TiO_2_ surface was modelled as a (001) slab in a 4- 1 × 1 (12-atom) cell. In the DFT modelling of Cu adhesion on TiO_2_, the 36-atom cells of (001) and (110) surfaces were used. There was at least a 25 Å vacuum between slabs, to prevent artificial electrostatic interactions.

All calculations were spin polarized. Hubbard correction (GGA + U) was used in a simplified version of Cococcioni and de Gironcoli’s work [29]. An effective U value of 3 eV for the Ti-d states was taken from the literature [30]. The k-point grid was sampled through a Monkhorst—Pack scheme [31], using 4 × 4 × 1 k-points. Electronic and ionic force convergence criteria were 10^−6^ Ry and 10^−4^ Ry/Bohr, respectively. The structures are presented in XcrysDen [32]. The charge of atoms was analyzed using Bader code [33].

The adhesion of metals was investigated at high-symmetry sites. The adhesion energy on the TiO_2_(001) surface was calculated according to Equation (1):

(1) $$E_{adh}=E_{surf+M}-E_{surf}-E_{M}$$

where E_surf+M_ is the total energy of the surface with adhered metal, E_surf_ is the total energy of the bare TiO_2_(001) or TiO_2_(110) surface and E_M_ is the total energy of the isolated metal atom.

### 2.2. Preparation of TiO_2_

TiO_2_ rutile nanopowder was prepared according to the procedure from [34]. Titanium (IV)–isopropoxide (Ti(OCH(CH_3_)_2_)_4_) (Sigma-Aldrich, St. Louis, MO, USA, 97%) was dissolved in isopropyl alcohol (Centrohem, Stara Pazova, Serbia, 99.5%) and stirred on a magnetic stirrer at room temperature. After a couple of minutes, TiO_2_ nanoparticles were precipitated with the addition of alkaline distilled water (pH 8). The reaction mixture was stirred at room temperature for 45 min. The molar ratio of alkoxide/alcohol/water was fixed at 5:3:1. The as-prepared precipitate was washed using deionized water, centrifuged, dried overnight in a drying oven at 100 °C, calcined at 700 °C for 5 h and left in the oven to cool down overnight.

### 2.3. Preparation of Cu/TiO_2_

Cu metal was deposited onto the TiO_2_ surface according to the method proposed in [35]. In total, 500 mg of prepared TiO_2_ nanoparticles was added to 50 mL of deionized water and dispersed using an ultrasonic bath at 90 °C for 30 min. Then, 5.8750 mg (0.4 molar % Cu compared to TiO_2_) of Cu(NO_3_)_2_·3H_2_O (Merck, Darmstadt, Germany, 99.5%) and 12.5 mg of solid NaOH (Lach-ner, Neratovice, Czech Republic, 99.6%) were added to the mixture and stirred for a couple of minutes. Next, 5 mL (50 mg/L) of a NaBH_4_ solution (BDH Chemicals Ltd., Poole, UK, 95%) was added drop-wise to the reaction mixture and stirred at room temperature for 1.5 h. Afterward, the suspension was washed using deionized water, centrifuged and, finally, dried overnight in the dryer at 100 °C.

### 2.4. Photodegradation of the Ciprofloxacin

The ciprofloxacin solution was prepared from a commercial ciprofloxacin–lactate solution for infusion (Marocen^®^, Hemofarm, Serbia), which contains 100 mg of the ciprofloxacin in 10 mL of the solution. First, the commercial solution was dissolved in deionized water in a volumetric flask of 250 mL and then 12.5 mL of this solution was dissolved in deionized water in another volumetric flask of 250 mL, with the final concentration of 4.75 × 10^−4^ M, 20 mg/L of ciprofloxacin.

The procedure of photodegradation was carried out in the same way for both photocatalysts. In total, 20 mg of finely powdered catalyst (TiO_2_ or (Cu)TiO_2_) nanoparticles was added into 50 mL of the ciprofloxacin solution (4.75 × 10^−4^ M, 20 mg/L of ciprofloxacin). The reaction mixture was stirred for 30 min in the dark, alongside a blank (solution of ciprofloxacin). Next, the blank and the mixture solutions were irradiated under UV light (Philips TUV 15W UVC Disinfection Lamps, Philips, Poland) for 4 h. Aliquots were taken after 0, 15, 30, 60, 120, 150, 180, 210 and 240 min for mixing and 30, 60, 120, 180 and 240 min for the blank in a 4 mL quartz cuvette. The photodegradation process was monitored with UV-Vis spectrometry (LLG Labware, Detroit, MI, USA), by recording the UV-Vis spectra in the wavelength range from 190 to 500 nm.

### 2.5. XRD Analysis

The crystal structure of TiO_2_-based powders was determined by analyzing X-ray powder diffraction (XRPD) data. The measurements were conducted on dried powders using a high-resolution SmartLab^®^ diffractometer (Rigaku, Japan), equipped with a Cu Kα radiation source (λ = 1.5406 Å) under a voltage of 40 kV and a 30 mA current. The data collection for the patterns was performed in the 10–70° 2θ range. The X-ray diffraction scan was conducted at a scan rate of 1°/min. The step size used during the scan was 0.02°. The phase identification of the synthesized materials as well as the crystallite size, lattice strain and lattice parameter were calculated using the Halder–Wagner method incorporated in PDXL2-integrated X-ray powder diffraction software (Version 2.8.40; Rigaku Corporation, Tokyo, Japan).

### 2.6. SEM Analysis

Scanning electron microscopy (SEM) with energy dispersive X-ray spectroscopy (EDS) was performed with a PhenomProX electron microscope (Phenom, Thermo Fisher Scientific, Waltham, MA, USA).

### 2.7. TOC Analysis

Total organic carbon (TOC) was measured on a TOC-LCPH analyzer (Shimadzu Co., Kyoto, Japan). Mineralization efficiency was calculated from Equation (2):

(2) $$Mineralization\;efficiency\left(\%\right)=\frac{1-{TOC}_{final}}{{TOC}_{initial}}\times 100$$

## 3. Results

### 3.1. DFT Screening of d-Metal Adhesion

In order to find the optimal metal coating for the TiO_2_ photocatalyst, DFT screening was performed for bare rutile TiO_2_(001) and (M)TiO_2_(001) for 24 transition metals (M) on three different binding sites: *hollow*, *bridge* and *top* (Figure 1).

DFT-calculated adhesion energies of transition metals on high-symmetry sites of the TiO_2_(001) surface are given in Table 1.

According to the obtained DFT calculation results, the *bridge* adsorption site is preferential for the majority of transition metals. All investigated metals show negative adhesion energies (i.e., adhesion is thermodynamically possible). Besides the E_adh_, the physical stability of the deposited overlayer also depends on the relation between E_adh_ and the cohesive energy as the intrinsic property of a metal (E_coh_). When E_adh_ > E_coh_, the metal is expected to form a stable monolayer, and vice versa, when E_coh_ > E_adh_, the metal is prone to form agglomerates [36].

**Figure 2:**
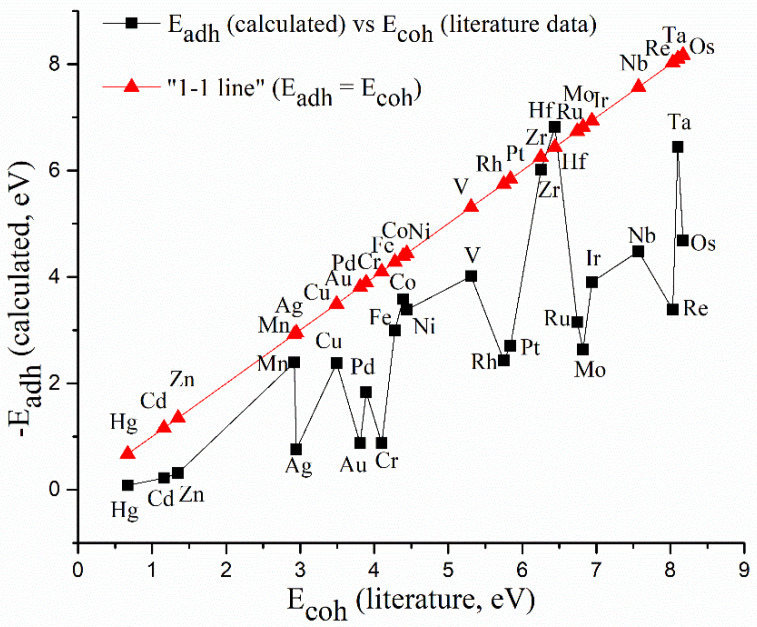
The correlation between metal cohesive energies from literature data (E_coh_) [36] and metal adhesion energies on TiO_2_(001) calculated in the present study (E_adh_) is represented by black squares. The “1-1 line” (E_adh_ = E_coh_), given for reference, is represented by red triangles. For such representations, all metals with E_adh_ < E_coh_ (weaker adhesion on TiO_2_(001) compared to cohesion) are below the “1-1 line”.

In Figure 2, calculated adhesion energies on preferential binding sites are correlated with experimental literature data on cohesive energies of metals, to predict their affinity to agglomerate on the TiO_2_ surface.

As can be seen in Figure 2, all d-elements except Hf exhibit lower calculated E_adh_ on the Ti(001) surface compared to literature experimental cohesive energies (E_adh_ < E_coh_). Therefore, the agglometration of all metals (except Hf) in a form of nanoparticles, rather than the formation of monolayers, is thermodynamically encouraged, making impossible the deposition of stable uniform overlayers at high metal loadings. On the other hand, in Figure 2, it can be seen that some metals—Hg, Cd, Zn, Mn, Cu, Fe, Co, Ni, V, Zr and Hf—still exhibit a smaller E_adh_-E_coh_ difference (are closer to the “1-1 line”) than the rest (Ag, Au, Cr, Rh, Pt, Mo, Ru, Ir, Nb, Re and Os). So, it was decided to select the model metal to be deposited from the first group, assuming that the smaller E_adh_-E_coh_ difference will additionally decrease the probability of metal agglomeration on the TiO_2_ surface.

Among the metals from the first group, Cu, affordable but noble, was selected as the most appropriate model metal to achieve our goal—to deposit a thin metal layer on the TiO_2_ surface and further study its photocatalytical behavior experimentally and theoretically. Namely, Cu can be easily reduced to the metallic state with the common NaBH_4_-based method, and is not prone to oxidation in aqueous media (see also Table S1). As calculated Cu adhesion energy (E_adh_ = −2.39 eV) is lower by 0.53 eV compared to the literature cohesive energy, it was decided to keep a low molar ratio (up to 0.5 molar % Cu vs. TiO_2_), in order to maximize the probability for Cu-TiO_2_ interaction and minimize the agglomeration of Cu particles.

**Figure 3:**
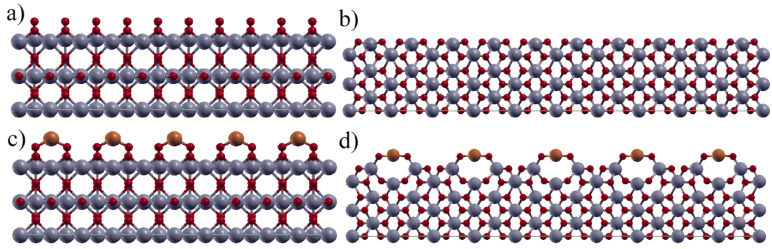
Optimized geometries of 36-atom slabs TiO_2_(001)—(**a**,**c**), and TiO_2_(110)—(**b**,**d**) with adsorbed Cu on bridge site. Color code: Ti—gray, O—red, Cu—brown.

To closer investigate the ability of Cu to bind on the TiO_2_ surface and further study the electronic properties of the system, the model is widened to the adhesion of a single Cu atom on (001) and (110) rutile planes in a larger, 36-atom cell. Optimized geometries of the investigated surfaces with and without adsorbed Cu are represented in Figure 3.

Calculated adhesion energies of Cu at the bridge site along with Bader charge transfer upon binding are given in Table 2.

The obtained values of adhesion energies confirm that the Cu atom is thermodynamically stable on the TiO_2_ rutile surface, and the binding is accompanied by a significant charge transfer from Cu to TiO_2_. Adhesion on TiO_2_(110) is significantly stronger compared to TiO_2_(001). Moreover, although agglomeration was initially expected based on DFT screening results, Cu binding with O(2) oxygens of the TiO_2_(110) surface results in strong ionic binding (E_adh_ > E_coh_) and one electron is completely transferred from Cu to TiO_2_.

In summary, obtained DFT results point out that, at low surface concentrations of Cu, a formation of a surface oxide is thermodynamically encouraged, due to the availability of the sites rich with unsaturated oxygen (a high surface energy). At higher surface concentrations of Cu, after the saturation of oxygen-rich sites, one might expect agglomerates of Cu atoms that are initially seeded at these sites.

**Figure 4:**
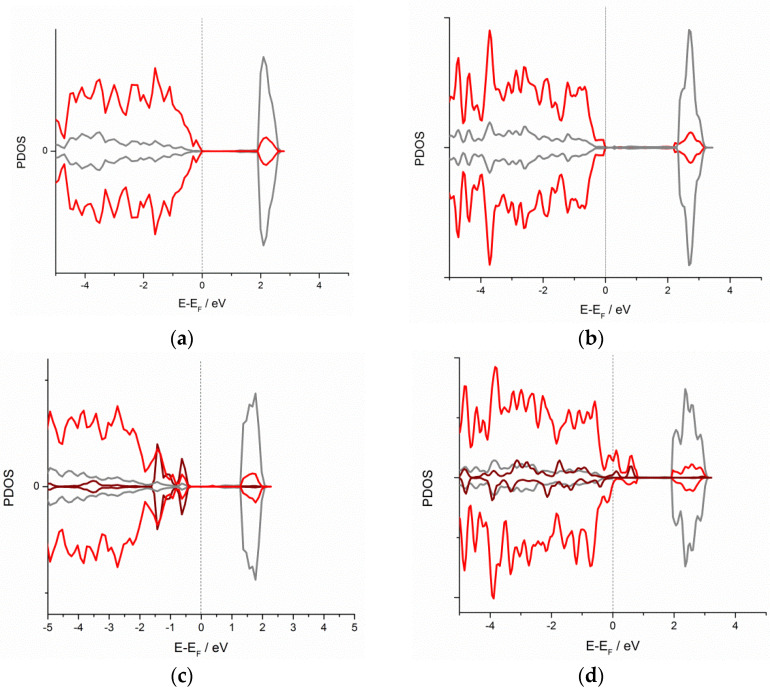
Electronic structure PDOS of (**a**) TiO_2_(001), (**b**) TiO_2_(110), (**c**) (Cu)TiO_2_(001) and (**d**) (Cu)TiO_2_(110) surfaces with and without adsorbed Cu. Fermi level is taken as energy zero. Color code: Ti states—gray, O states—red, Cu states—wine.

The electronic structure projected density of states (PDOS) of TiO_2_(001) and TiO_2_(110) with and without adsorbed Cu is represented in Figure 4.

PDOS structures essentially confirm the formation of the Cu-O bond. In both cases of (001) and (110) surfaces, Cu features appear on the top of the valence band, overlapping with O states. Although the quantitative representation of a bandgap with DFT requires artificially large U-correction [37], the decrease in a bandgap width upon the introduction of novel states is clearly visible. Also, in the case of the (001) surface, there is a downshift of the Ti d-band upon Cu adsorption, pointing to the partial contribution of the metal bond, while on the (110) surface, there is no significant downshift of the Ti d-band, pointing to that the newly formed Cu-O bond is barely ionic.

### 3.2. Characterization of Prepared Photocatalysts

**Figure 5:**
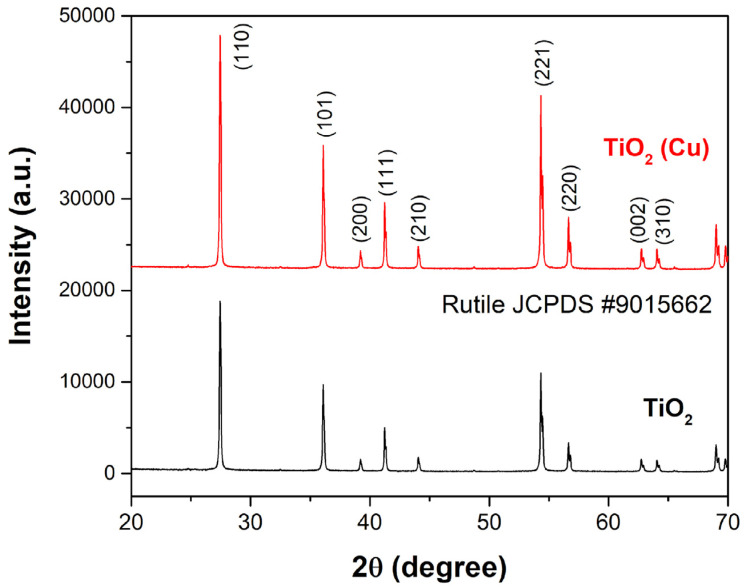
XRD patterns of freshly prepared (Cu)TiO_2_ and bare TiO_2_ powders.

XRD patterns of prepared (Cu)TiO_2_, compared to bare TiO_2_, are presented in Figure 5.

The XRPD pattern of (Cu)TiO_2_ is remarkably similar to the one of bare TiO_2_. Such a result is expected, as the initial amount of Cu in the (Cu)TiO_2_ sample preparation is below the detection limit of the crystallographic method. The good agreement with JCPDS #9015662 points out that the prepared materials crystallize in the tetragonal *P*4_2_/*mnm* space group as a pure rutile phase of titanium dioxide. The patterns also show that the crystalline structure of TiO_2_ remains stable after the modification with the Cu coating. The crystallite size, lattice strain and lattice parameter calculated with the Halder–Wagner method are listed in Table 3. As can be seen, there are only slight differences in the crystalline properties of these two materials, whereby the crystallites are slightly larger in TiO_2_(Cu).

**Figure 6:**
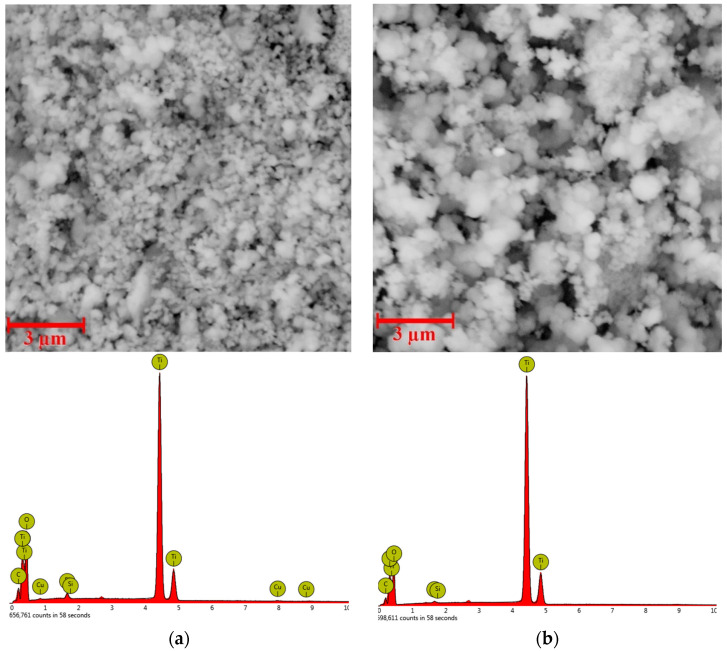
SEM images and EDX spectra of (**a**) (Cu)TiO_2_ and (**b**) bare TiO_2_ at the magnification ×20,000.

SEM images and EDX spectra of prepared (Cu)TiO_2_ and bare TiO_2_ samples are represented in Figure 6.

SEM images (Figure 6) of bare TiO_2_ reveal variable-shape agglomerates of spherical nanoparticles of about 1 µm in diameter. The (Cu)TiO_2_ sample exhibits a similar structure, although the average size of agglomerates is lower compared to bare TiO_2_. The average diameter of nanospheres in (Cu)TiO_2_ is also lower compared to bare TiO_2_ (about 100 nm), being comparable with an average crystallite size obtained from a Debye–Scherrer analysis.

**Figure 7:**
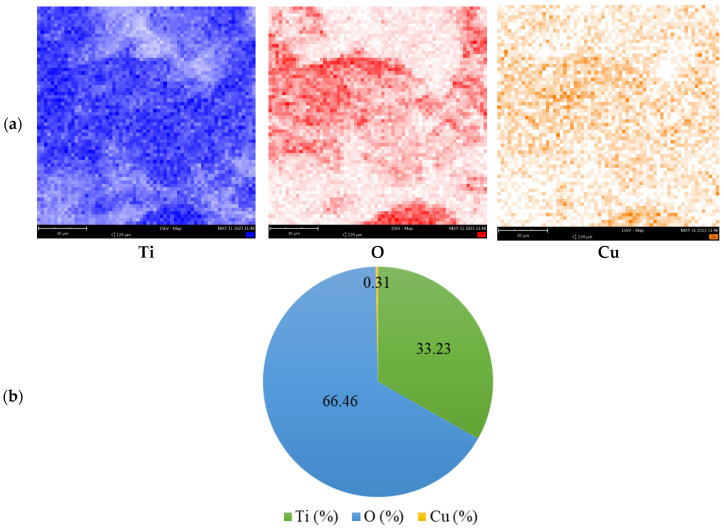
(**a**) Elemental maps of Ti, O and Cu in (Cu)TiO_2_ sample. (**b**) Normalized amount of the elements from EDS analysis.

The EDX pattern (Figure 6) confirms the presence of Cu in the (Cu)TiO_2_ sample. Moreover, elemental maps of (Cu)TiO_2_ (Figure 7) confirm that copper and oxygen follow similar and rather uniform spatial distribution—oxygen-rich areas are also rich in copper—confirming the preposition of prevalent Cu-O binding at low Cu loadings from DFT calculations. Si peaks, present in both bare and Cu-coated samples, can be attributed to SiO_2_ as a residual impurity, probably from calcination, while detected carbon originates from the carbon-based support for SEM imaging and Ti-isopropoxide residues from synthesis.

The normalized amount (Figure 7b) of Cu in the (Cu)TiO_2_ sample is 0.31 atomic %, being close to the input amount of Cu during sample preparation (0.40%).

### 3.3. Photodegradation of Ciprofloxacin

**Figure 8:**
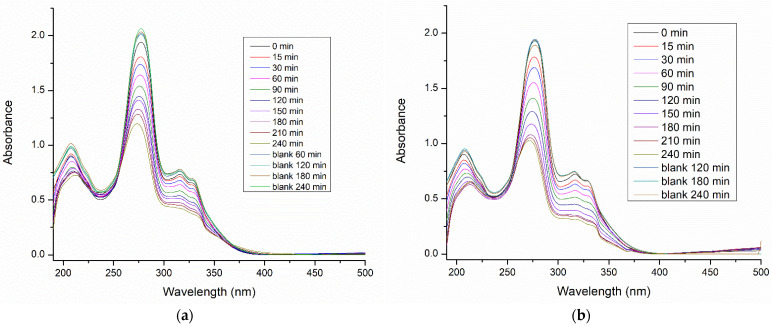
UV-Vis spectra of the ciprofloxacin solution of the initial concentration 4.75 × 10^−4^ M during 240 min of photodegradation on bare TiO_2_ (**a**) and (Cu)TiO_2_ (**b**).

Prepared (Cu)TiO_2_ was applied as a photocatalyst for the degradation of ciprofloxacin. UV-Vis spectra of 4.75 × 10^−4^ M of a ciprofloxacin solution during 240 min of photo-treatment with (Cu)TiO_2_, compared with bare TiO_2_, are presented in Figure 8.

UV-Vis spectra confirm that the characteristic absorption maximum of ciprofloxacin at about 277 nm decreases with time, due to the photodegradation. UV-Vis spectra of bare TiO_2_ and (Cu)TiO_2_ photocatalysts (Figure S2) confirm that the investigated photocatalysts do not absorb on the area of interest for tracking ciprofloxacin degradation.

The degradation rate was calculated from the decrease in absorbance at 277 nm, assuming the pseudo-first-order kinetics (Equation (3)):

(3) $$A=A_{0}\cdot e^{-kt}$$

where A_0_ is the absorbance at time *t* = 0, and *k* (min^−1^) is a pseudo-first-order rate constant.

The linearized form of Equation (3) is given in Equation (4).

(4) $$ln\left(\frac{A}{A_{0}}\right)=-kt$$

was applied to obtain the rate constant *k* from the slope of the graph.

**Figure 9:**
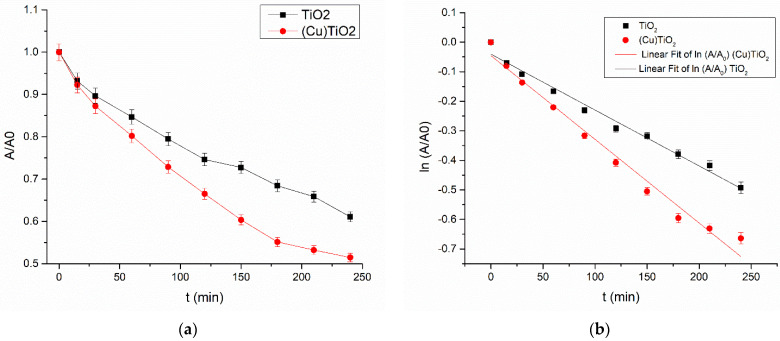
Degradation curves (**a**) and their linearized pseudo-first-order fits (**b**) of ciprofloxacin degradation on bare TiO_2_ (black squares) and (Cu)TiO_2_ (red circles).

Resulting kinetic curves of CIP photodegradation on (Cu)TiO_2_ and bare TiO_2_ photocatalysts are represented in Figure 9.

Calculated pseudo-first-order rate constants are given in Table 4.

As is evident from Figure 8, about 37% of ciprofloxacin was degraded within 240 min in the presence of the bare TiO_2_ photocatalyst, and the degradation extent was raised to about 50% in the presence of (Cu)TiO_2_ within the same time. In Table 4, it is confirmed that the degradation rate on (Cu)TiO_2_ is significantly (about 1.5 times) higher compared to non-modified TiO_2_. The mineralization extent was measured with a TOC analysis at the end of the degradation process, and the results are represented in Table 5.

As can be seen from Table 5, the spectrometrically detected ciprofloxacin degradation is followed by the mineralization of the organic matter in the sample. The total mineralization extent is slightly larger for (Cu)TiO_2_—12.2%—compared to bare TiO_2_ (9.5%).

## 4. Discussion

In order to identify the acceptable metals to prepare a thin metal layer of controllable properties on a TiO_2_ photocatalyst, comprehensive DFT screening of the adsorption of d-metals on TiO_2_(001) was carried out. However, due to the lower affinity for adsorption than for agglomeration (E_adh_ < E_coh_), almost all investigated metals showed similar expected behavior, with the thermodynamically favorized agglomeration of metal particles, pointing out that the metal concentration, rather than the type, is a major factor determining its structure on the TiO_2_ surface. Accordingly, it was concluded that keeping a small amount of metal coating (up to 0.5%) will help in avoiding excessive metal agglomeration. Among investigated metal adsorbates, Cu was found to be reasonably thermodynamically stable, available and easy to prepare, so it was selected as a model metal for further work.

However, subsequent and more detailed calculations revealed that adsorption is still thermodynamically favorized on the oxygen-rich sites, yielding in a formation of some kind of surface oxide. The widened DFT model predicted the strong interaction of Cu with non-saturated oxygens on TiO_2_(110) (E_adh_ > E_coh_) followed by a considerable charge transfer from Cu to TiO_2_. The electron transfer from the metal to semiconductor is, at first glance, somewhat surprising, as one might expect a charge transfer in the opposite direction due to the formation of the Schottky junction [38]. However, the obtained result is in good agreement with the literature data on the deposition of other metal atoms, such as Pt and Pd [30] and Au [39], as well as with experimental XPS data that confirm the presence of Cu-O on the surface of Cu-modified TiO_2_ [40]. In addition, it was shown with earlier theoretical studies of similar systems that the charge transfer from a semiconductor to metal is promoted by oxygen vacancies and is also dependent on metal loading, i.e., on metal cluster size [41]. The PDOS calculations revealed that, upon the introduction of Cu, novel states appear on the top of the valence band, and thus the bandgap width is decreased, confirming the possibility of a synergistic effect of TiO_2_ and deposited Cu to build an interface with improved optical properties for photocatalytic processes.

In the next step, Cu-coated TiO_2_ was produced using the NaBH_4_ chemical reduction method, and it was then used to photodegrade the antibiotic ciprofloxacin. The XRD analysis confirmed that the rutile TiO_2_ was successfully prepared and stable after the deposition of the Cu coating. The findings of the SEM imaging showed that the agglomeration of TiO_2_ particles is reduced in the presence of the Cu coating, as the size of nanoparticle agglomerates is reduced from about 1 μm to approximately 100 nm, being comparable with the average crystallite size from the Debye–Scherrer analysis (91 ± 5 nm). The obtained reduction in particle size points to that the adsorbed Cu probably impedes the agglomeration of TiO_2_ and thus contributes to the larger TiO_2_ surface area available for the photocatalytic process. The EDX analysis confirmed the presence of uniformly distributed Cu in the prepared sample, following the similar spatial distribution as oxygen, and thus additionally confirming its binding affinity towards oxygen. A small loss of Cu during synthesis (0.31% EDX-detected compared to 0.40% input) is in good agreement with the expected good stability of the Cu coating, predicted from DFT calculations. The results are also comparable with TEM findings of Eskandarloo et al. [35], where Cu was identified as 10 nm dots deposited onto TiO_2_ nanospheres of about 50 nm in diameter.

The newly prepared photocatalyst showed an improved performance for the degradation of ciprofloxacin compared to bare TiO_2_ rutile. The degradation rate increased by a factor of 1.5. The obtained improvement in the degradation performance of the modified photocatalyst is expected considering the reduced agglomeration and smaller TiO_2_ particle size in the presence of the Cu coating.

The current work is compared with data from similar studies in Table 6.

The results are in reasonable agreement with the available literature data on similar catalytic materials, considering the low amount of the catalyst compared to the pollutant concentration in the present study. Although the increase in the catalyst amount would certainly further increase the reaction rate, high catalyst loadings are avoided in this study in order to assure the reliable detection of ciprofloxacin with UV-Vis spectrometry.

The DFT screening of adhesion energies of d-metals appeared to be a reasonable strategy for the improvement in efficiency of TiO_2_-based photocatalysts. Efficient metal binding at low loadings contributes to the better availability of the photocatalyst surface, due to the less pronounced agglomeration of TiO_2_ particles. Moreover, the real chemical binding of metals on surface sites rich in energy, which can be successfully tracked using DFT calculations, does contribute to the formation of novel synergistic structures that modify the intrinsic optical properties of input materials, having a perspective to further tune the photocatalytic performances.

## Figures and Tables

**Table 1:** Adhesion energies (E_adh_) of investigated metals on high-symmetry sites of TiO_2_(001) surface (n.s. stands for “non-stable”).

Metal/Ads. Site	Bridge (eV)	Hollow (eV)	Top (eV)	Metal/Ads. Site	Bridge (eV)	Hollow (eV)	Top (eV)
Ag	−0.868	−0.690	−0.274	Nb	−4.476	−4.416	−0.604
Au	−0.680	−0.757	−0.523	Ni	−3.376	−2.967	n.s.
Cd	−0.220	n.s.	−0.050	Os	−4.675	−3.902	−0.675
Co	−3.581	−2.967	−0.422	Pd	−1.398	−1.830	−0.806
Cr	−0.870	n.s.	−0.115	Pt	−1.962	−2.698	−0.939
Cu	−2.376	−1.206	−0.270	Re	−2.700	−3.382	−0.259
Fe	−2.492	−2.994	−0.431	Rh	−2.429	−1.802	−0.799
Hf	−6.817	−2.695	−1.106	Ru	−2.805	−3.145	−0.860
Hg	−0.076	−0.048	−0.041	Ta	−4.805	−6.435	−0.769
Ir	−2.887	−3.897	−0.979	V	−4.006	−2.348	−0.342
Mn	−2.385	−0.931	−0.268	Zn	−0.314	−0.077	−0.053
Mo	−2.628	−2.598	−0.141	Zr	−6.014	−5.503	−1.170

**Table 2:** Calculated adhesion energies of Cu and electron transfer.

	E_adh_ (eV)	Electrons Transferred from Cu to TiO_2_
(Cu)TiO_2_(001)	−2.61	0.65
(Cu)TiO_2_(110)	−6.71	1.05

**Table 3:** Crystallite size, lattice strain and lattice parameter of the prepared TiO_2_-based materials.

Sample	Crystallite Size (Å)	Lattice Strain (%)	Lattice Parameter (Å)
TiO_2_(Cu)	751(19)	0.01(3)	*a* = 4.59332(8) *b* = 4.59332(8) *c* = 2.95910(7)
TiO_2_	685(20)	0	*a* = 4.59274(14) *b* = 4.59274(14) *c* = 2.95958(12)

**Table 4:** Calculated pseudo-first-order rate constants of CIP degradation on TiO_2_ and (Cu)TiO_2_ photocatalysts.

	k (min^−1^)	R^2^
(Cu)TiO_2_	0.0028 ± 0.0002	0.97942
TiO_2_	0.00189 ± 0.00009	0.98365

**Table 5:** Results of TOC analysis.

	Initial TOC (t = 0) mg/L	Final TOC (t = 240 min) mg/L	TOC Removal (%)
TiO_2_	14.8	13.4	9.5
(Cu)TiO_2_	14.8	13.0	12.2

**Table 6:** Comparison of current work to literature data.

Catalyst	Catalyst Dosage (mg/L)	Initial Concentration (mg/L)	UV-Irradiation Parameters	Time (min)	Removal Efficiency (%)	Reference
Cu/TiO_2_	20	20	15 W λ = 254 nm	240	50	Present study
TiO_2_	700	80	24 W λ = 254 nm	600	89	[1]
TiO_2_	120	20	Irradiance, 100 Wm^−2^ λ = 365 nm	60	75	[42]
GMC-TiO_2_ composite	350	15	14 W λ = 254 nm	90	100	[43]
Cu-doped AC/TiO_2_	320	100	7 W λ = 254 nm	120	95	[44]
TiO_2_/MMT	100	20	16 W UV-C	120	62	[45]

## Data Availability

Not applicable.

## Supplementary Materials

The following supporting information can be downloaded at: https://www.mdpi.com/article/10.3390/ma16165708/s1, Figure S1. Structure of ciprofloxacin [46]; Table S1: Qualitative scoring table for metal selection; Figure S2. UV-Vis absorption spectra of bare TiO_2_ and TiO_2_(Cu).
