# Supplementary material for: In Silico Guided Design of Metal/Semiconductor Photocatalysts: A Case of Cu-Modified TiO2 for Ciprofloxacin Degradation
Source: Materials (Basel). 2023 Aug 21;16(16):5708. doi: 10.3390/ma16165708 (PMC10456727; doi:10.3390/ma16165708)
Supplement: Supplementary file 1 [file materials-16-05708-s001.zip › materials-2538708-supplementary.pdf]

Supplementary material to

In silico guided design of metal/semiconductor photocatalysts: a case of Cu-modified TiO_2 for ciprofloxacin degradation

Marija Kovačević[1], Sanja Živković[1], Miloš Ognjanović[1], Miloš Momčilović[1], Dubravka Relić[2], Dragana Vasić Anićijević[1*]

[1] University of Belgrade, Vinča Institute of Nuclear Sciences-National Institute of the Republic of Serbia, Mike Petrovića Alasa 12-14, Belgrade, Serbia
[2] University of Belgrade, Faculty of Chemistry, Studentski trg 12-14 Belgrade, Serbia
* draganav@vin.bg.ac.rs

S1. Ciprofloxacin info

Ciprofloxacin (CIP), a fluoroquinolone antibiotic, is used to treat a variety of bacterial infections, including those of the lungs and the urinary tract (Figure 1) [1]. Being commonly prescribed in different pharmaceutical forms (pills, i.e., infusion solution, eyedrops), it is widely present in wastewater, medical and pharmaceutical waste. The CIP ingested by humans or animals is partially metabolized, leading to significant partial excretion into the environment in the pharmacological form. Thus, high concentrations of CIP can persist in feces up to several days after the treatment is finished.

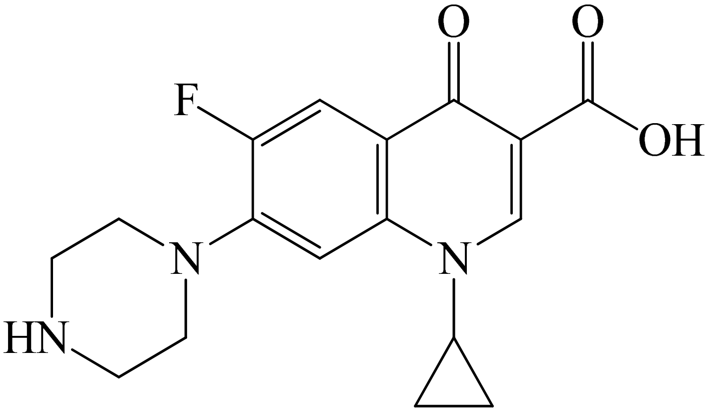

Figure S1. Structure of ciprofloxacin

Table S1: Qualitative scoring table for metal selection

	Metal	Affordable	Highly Toxic	Easily oxidized	Easy to deposit
1st group	Hg	+	+	-	+
	Cd	+	+	+	+
	Zn	+	-	+	-
	Mn	+	+	+	-

	**Cu**	+	-	-	+
	**Fe**	+	-	+	+
	**Co**	+	-	+	-
	**Ni**	+	-	+	-
	**V**	-	+	+	-
	**Zr**	-	-	+	-
	**Hf**	-	**unknown**	+	-
**2nd group**	**Ag, Au, Cr, Rh, Pt, Mo, Ru, Ir, Nb Os and Re**	Eliminated due to higher E_{coh}-E_{ads} difference compared to the first group			

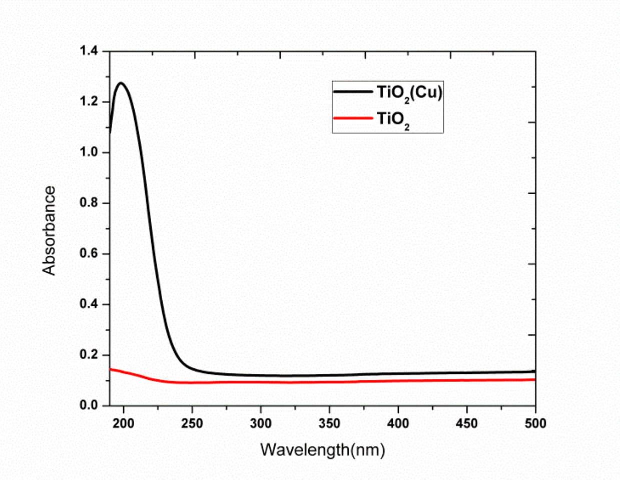

Figure S2. UV-Vis absorption spectra of bare TiO_2 and TiO_2(Cu) in aqueous medium at concentration 50 mg/L.

References

1. Thai, T.; Salisbury, B.H.; Zito, P.M. *Ciprofloxacin, in StatPearls*; StatPearls Publishing: St. Petersburg, FL, USA, 2023
